# Age of onset in chronic diseases: new method and application to dementia in Germany

**DOI:** 10.1186/1478-7954-11-6

**Published:** 2013-05-02

**Authors:** Ralph Brinks, Sandra Landwehr, Regina Waldeyer

**Affiliations:** 1 Institute for Biometry and Epidemiology, German Diabetes Center, Auf’m Hennekamp 65, Duesseldorf, 40225, Germany; 2 Department of Public Health, Heinrich Heine University, Duesseldorf, 40225, Germany

## Abstract

**Background:**

Age of onset is an important outcome to characterize a population with a chronic disease. With respect to social, cognitive, and physical aspects for patients and families, dementia is especially burdensome. In Germany, like in many other countries, it is highly prevalent in the older population and imposes enormous efforts for caregivers and society.

**Methods:**

We develop an incidence-prevalence-mortality model to derive the mean and variance of the age of onset in chronic diseases. Age- and sex-specific incidence and prevalence of dementia is taken from published values based on health insurance data from 2002. Data about the age distribution in Germany in 2002 comes from the Federal Statistical Office.

**Results:**

Mean age of onset of a chronic disease depends on a) the age-specific incidence of the disease, b) the prevalence of the disease, and c) the age distribution of the population. The resulting age of onset of dementia in Germany in 2002 is 78.8 ± 8.1 years (mean ± standard deviation) for men and 81.9 ± 7.6 years for women.

**Conclusions:**

Although incidence and prevalence of dementia in men are not greater than in women, men contract dementia approximately three years earlier than women. The reason lies in the different age distributions of the male and the female population in Germany.

## Introduction

Worldwide, dementia is a major public health problem today and in the future. The current number of cases is estimated to be 35.6 million, about one-fifth of those living in Western Europe [1]. In Germany, the country with most inhabitants in Europe, the number of cases will likely double by 2050 [1]. Patients with dementia encounter a variety of limitations including social, cognitive, psychological, and physical aspects with substantial loss of quality of life for the patients themselves and also for caregivers and families [2]. The economic impact of dementia is enormous. Associated annual costs are estimated at 604 billion US dollars worldwide and will increase even more quickly than the prevalence [1].

Age of onset of a disease has been described as an alternative to incidence as a measure for occurrence and effect in epidemiology [3]. Traditionally, comparisons between groups with a factor present or absent are expressed as relative risks. In common diseases with a high background risk, rate ratios between groups (i.e., ratios of person-time incidence rates) cannot be interpreted as risk ratios. In these cases, a statement that someone being exposed to a risk factor contracts the disease, on average, a number of years earlier than someone who is not exposed, is easily interpretable to nonepidemiologists [3]. In decisions of policy-makers, such as the planning of the need for special care units and nursing homes, the age of onset can be seen as a key measure. With respect to dementia, the age of onset is hardly accessible by empirical studies. In Germany, registers of newly diagnosed cases do not exist, and representative surveys of the age of onset are difficult to conduct. Besides presenting a feasible, new way of estimating the mean age of onset of a chronic disease, this article shows that age of onset depends on the age distribution of the population under consideration.

Assuming that the age-course of the incidence is known, we use a simple incidence-prevalence-mortality (IPM) model for calculating the mean age of onset of the chronic disease. In a first step, the general IPM model is introduced. Then, formulas for the age of onset will be developed and will be applied to epidemiological data on dementia.

## Methods

**Figure 1:**
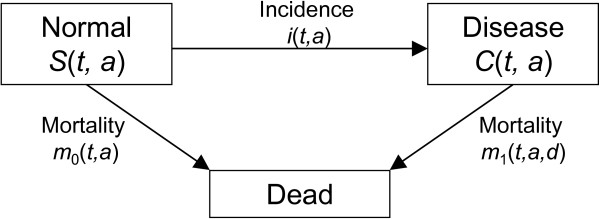
**IPM model.** Simple model of a chronic disease with three states. People in the state *Normal* are healthy with respect to the considered disease. In the state *Disease* they suffer from the disease. The transition rates depend on the calendar time *t*, on the age *a*, and in the case of mortality *m*_1_ of the diseased people, on the disease’s duration *d*.

In consideration of basic epidemiological parameters such as incidence of, prevalence of, and mortality from a disease, it is helpful to look at state (or compartmental) models. The model used here consists of the three states *Normal*, *Disease*, and *Death* and the transitions between the states. *Normal* means healthy with respect to the disease under consideration. The numbers of people in the *Normal* state are denoted as *S* (susceptible), while in the *Disease* state they are denoted as *C* (cases). The transition rates are the incidence rate *i* and the mortality rates *m*_0_ and *m*_1_ of the nondiseased and diseased people, respectively (Figure 1).

### Age of onset in the IPM model

In the general IPM model, the rates depend on calendar time (*t*), age (*a*), and in the case of *m*_1_, the disease duration (*d*). For a specific point in time t* and a small time period Δ > 0, the number of newly diseased people aged *a* is about *i*(t*, *a*) *S*(t*, *a*) Δ. By integration we obtain the number of all newly diseased people at time t* across all ages:

(1) $$\int_{0}^{w}\;i\left(t^{*},a\right)\;S\left(t^{*},a\right)\;da.$$

The upper limit *w* in Equation (1) is the age of the oldest member in the population. The mean age of onset $\bar{A}\left(t^{*}\right)$ at time t* is obtained by weighting the integrand in Equation (1) with the age *a* and dividing by the number of all newly diseased people. Hence it holds

(2) $$\bar{A}\left(t^{*}\right)=\frac{\int_{0}^{w}\;a\;i\left(t^{*},a\right)\;S\left(t^{*},a\right)\;da}{\int_{0}^{w}\;i\left(t^{*},a\right)\;S\left(t^{*},a\right)\;da}.$$

In practical applications the number *S* of nondiseased subjects in a population is not accessible. By setting *N*(t*, *a*) = *S*(t*, *a*) + *C*(t*, *a*) and *p*(t*, *a*) = *C*(t*, *a*)/*N*(t*, *a*), it holds *S*(t*, *a*) = {1 – *p*(t*, *a*)} *N*(t*, *a*). The function *N*(t*, *a*) is the (absolute) age distribution of the population, and *p*(t*, *a*) is the age-specific prevalence at time t*. Then, Equation (2) reads as

(3) $$\bar{A}\left(t^{*}\right)=\frac{\int_{0}^{w}\;a\;i\left(t^{*},a\right)\;\left\{1-p\left(t^{*},a\right)\right\}\;N\left(t^{*},a\right)\;da}{\int_{0}^{w}i\left(t^{*},a\right)\;\left\{1-p\left(t^{*},a\right)\right\}\;N\left(t^{*},a\right)\;da}.$$

Mostly, the age distribution *N* can be obtained from official vital statistics of the population under consideration. The incidence *i* and the prevalence *p* in Equation (3) is subject to epidemiological studies.

By interpreting the mean age at onset as the first moment of a random variable *A*(t*), the corresponding variance is

(4) $$\mathrm{Var}\left(A\left(t^{*}\right)\right)=\frac{\int_{0}^{w}\;{\;\left(a-\bar{A}\left(t^{*}\right)\right)}^{2}\;i\left(t^{*},a\right)\;\left\{1-p\left(t^{*},a\right)\right\}\;N\left(t^{*},a\right)\;da}{\int_{0}^{w}i\left(t^{*},a\right)\;\left\{1-p\left(t^{*},a\right)\right\}\;N\left(t^{*},a\right)\;da}.$$

Equations (3) and (4) hold true for subpopulations as well. In many diseases, the incidence *i*, the prevalence *p*, and the age distribution *N* differ substantially between sexes. Thus, it may be useful to apply Equations (3) and (4) to males and females separately.

### Relations between incidence, prevalence, and mortality

Besides the age distribution *N*, Equations (3) and (4) depend on the incidence *i* and the prevalence *p*. In cases where one of *i* or *p* is unknown, it may be possible to approximate it. For this, we assume that the transition rates do not depend on *t* or on *d*. In this situation Murray and Lopez considered a system of ordinary differential equations (ODEs), which expresses the change in the numbers of healthy and sick patients aged *a* with the corresponding rates [4,5]. The system can be transformed into a scalar ODE of Riccati type [6]:

(5) $$\mathrm{dp}/\mathrm{da}=\left(1-p\right)\;\cdot \;\left\{i-\mathrm{p\cdot}\;\left(m_{1}-m_{0}\right)\right\}.$$

This equation relates the change in the prevalence at age *a* to the rates *i*, *m*_0_, and *m*_1_. The advantage of such closed-form ODEs includes the possibility of calculating the age profile of the prevalence from given age-specific incidence and mortality rates. Under certain smoothness constraints, the incidence and mortality rates uniquely determine the prevalence. In addition, for given prevalence and mortality rates the incidence can be obtained, which allows cross-sectional studies to be used for incidence estimates [6].

### Application: dementia in Germany

The formulas developed above are applied to epidemiological data on dementia in Germany. The age-specific incidence has been taken from German health insurance data, separately for males and females [7]. The data have been interpolated affine-linearly using the middle of the age classes as knots. The mortality *m* of the general population is taken from the life tables of the Federal Statistical Office of Germany [8]. The reference year t* is 2002. The relative mortality *R* = *m*_1_/*m*_0_ is set constant to *R* = 2.4 [9]. Although it is likely that *R* depends on *a*, the age-specific values are not reported [9]. In case the general mortality *m* = (1 – p) · *m*_0_ + *p* · *m*_1_ and the relative mortality *R* are given, the ODE (2) changes its type and becomes Abelian [10]:

(6) $$\mathrm{dp}/\mathrm{da}=\left(1-p\right)\;\cdot \;\left\{i–\mathrm{m\cdot p\cdot}\;\left(R–1\right)/\left[\mathrm{p\cdot}\;\left(R–1\right)+1\right]\right\}.$$

The age-specific prevalence for men and women is derived by integrating the Abelian ODE (6) with initial condition *p*(60) = 0 via the classical Runge-Kutta method, cf. [11]. With *N*(2002, *a*) given for every age *a* = 0, …, 99, 100+ from the official statistics [9], the integrals in Equations (3) and (4) are replaced by sums. All calculations are performed with the Software R (The R Foundation for Statistical Computing), version 2.12.0.

## Results

**Figure 2:**
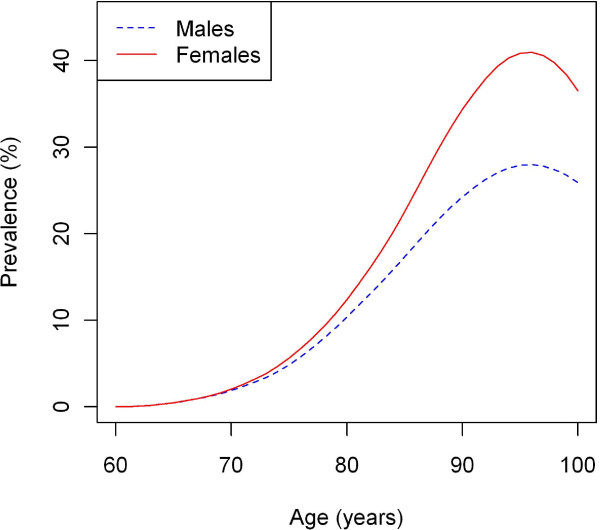
**Prevalence of dementia in Germany.** Age- and sex-specific prevalence of dementia after integration of the ODE (6).

Figure 2 shows the age-specific prevalence of dementia in Germany for males and females. In both cases, the prevalence starts at 0 at the age of 60 years, which is the initial condition. Until the age of 70 years, the prevalences of dementia in men and women are almost identical. Then the curves start to diverge, which is an effect of the difference in general mortality. Incidence rates in this age class are almost the same for men and women. However, general mortality *m* for men is almost twice as high as for women. It is striking that both prevalence curves have a maximum at age a* = 96 years. At this age it holds d*p*/d*a* = 0.

**Figure 3:**
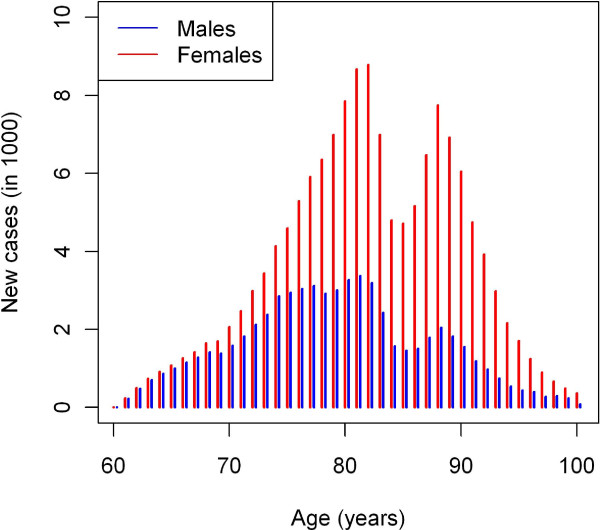
**Age distribution of incident dementia.** Age distribution of the number of new cases (blue: males, red: females) in 2002 based on the age distribution *N*(2002, *a*) in Germany.

The age distribution of the new cases of dementia *i*(t*, *a*) · (1 – *p*(t*, *a*)) · *N*(t*, *a*) for each age *a* = 60, …, 99, 100+ at t* = 2002 is shown in Figure 3 for males and females. Both distributions are left-skewed. The discontinuities stem from the discontinuous structure of the age distribution. Women are far more often affected than men. The modus of the age of new cases is at age 80 and 85 in men and women, respectively.

The associated mean age of onset $\bar{A}\left(t^{*}\right)$ of dementia together with the standard deviation are presented in Table 1. On average, males contract the disease at the age of 78.8 years, whereas females develop it three years later at 81.9 years of age. The standard deviation of the age of onset is similar in men and women at about 8 years. The source and data files for calculating these numbers using the R software are provided as Additional file 1 to this article.

## Discussion

The framework of the IPM model allows the calculation of the mean age of onset of a chronic disease. One might expect that the age at onset only depends on the disease, that it is disease inherent. However, the age at onset depends on the shape of the age distribution. The age distribution is a subject of demography, and there are population models where the numbers of people in the age groups can be represented analytically. The simplest example is the *stationary population*[12]. However, real populations typically are nonstationary and have to be managed differently. In Germany, like in many other countries, the age structure of the population is captured accurately by the Federal Statistical Office.

**Table 1:** Onset and duration of dementia for males and females in Germany in 2002

	**Age of onset (years)**	
	**Mean**	**Standard deviation**
Males	78.8	8.1
Females	81.9	7.6

**Figure 4:**
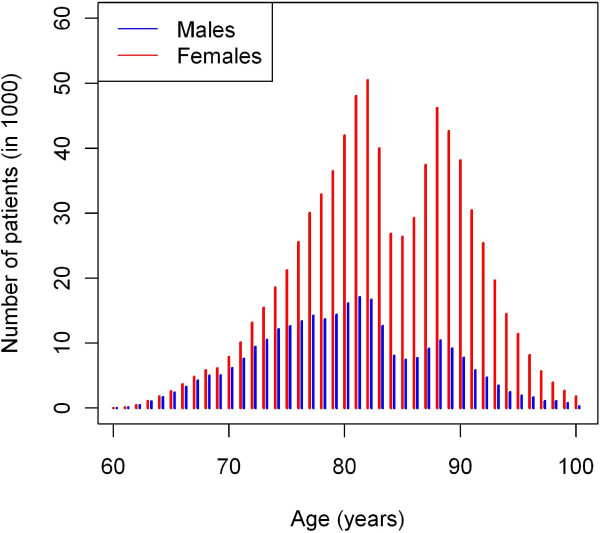
**Age distribution of people with dementia.** Age distribution of the number of people with dementia (blue: males, red: females) in 2002 based on the age distribution *N*(2002, *a*) in Germany.

When applying the methods to dementia in Germany, the mean age of onset of dementia is about 79 in men and 82 in women. Due to the different life expectancies of men and women in Germany, there are far more females in the older age groups, and the difference is not surprising. It is clear that there is a large difference in the numbers of male and female patients with dementia. Figure 4 shows the numbers of female and male patients in each of the age groups. In 2002, a total of about 63,000 men and 147,000 women aged 60 years and above fell ill. The reasons for this discrepancy between men and women are twofold. First, the incidence of dementia is higher in females, which leads to a higher prevalence (see Figure 2). Second, the number of individuals over 60 years is higher in females. For comparison, there were only 8.5 million men and 11.6 million women 60 years and above in Germany in 2002 [8].

Another point is worth being mentioned: the examinations in this paper predict a peak in the prevalence of dementia in the second half of the ninth decade of life for men and women. After the peak, prevalence decreases. In another survey from 2007 about the prevalence of dementia in Germany, there are indications of the existence of a maximum in the age-specific prevalence [13].

The analytical representations of the mean age of onset and have several advantages. First, by the formulas (2) – (4) the effects of interventions in chronic diseases on $\bar{A}\left(t^{*}\right)$ can be estimated in advance. For example, if a prevention program lowers the incidence of the disease by a certain amount, the prevalence is lowered [6] and the impact of the incidence reduction on $\bar{A}\left(t^{*}\right)$ can be predicted. Second, by making the dependence of $\bar{A}\left(t^{*}\right)$ on the age distribution explicit, the necessity of proper age profiles (or adjustment methods) in epidemiological studies that survey age of onset becomes obvious. With respect to surveying the age of onset empirically (e.g., by questioning patients about their age at date of diagnosis), Chen et al were aware of the problem of choosing a representative age distribution and gave some corresponding advice [14]. Finally, the approach presented for the first time in this article allows the estimation of the mean age of onset of dementia in the entire relevant population of Germany. Currently, there are no patient registers about dementia in Germany, and surveys involving patients with dementia and relatives are very difficult.

Nevertheless, this work has some weaknesses. First, our way of calculating the age-specific prevalence of dementia for men and women requires the incidence and mortality rates to be independent from calendar time and independent from disease duration. These independence assumptions are hardly fulfilled in real data. In most populations, mortality has a secular trend due to medical progress and health awareness. Similarly, in the calculation of prevalence, the relative mortality is assumed to be constant for all age groups and both sexes. This is unlikely to be true, but more detailed data are lacking. However, the age- and sex-specific prevalences are quite similar to another survey [13], which gives justification for our method. Second, the age distribution of the Federal Statistical Office does not stratify ages beyond 100 years. The people 100 years and above are summarized in one age class 100+. With a view to the relatively low case numbers (see Figure 3), the effect of this limitation is negligible. Third, the incidences are based on claims data of the statutory health insurance (SHI) from 2002. Therefore, age of onset actually means age of diagnosis. Furthermore, there is a large proportion of undetected cases in dementia [15,16], which are not considered in the present study. Additionally, the rate of officially diagnosed cases may depend on formal or reimbursement reasons or the sensibility for the disease.

## Conclusions

In the present work, an IPM model has been used to study the age of onset of dementia. The mean age of onset depends on the incidence and prevalence of the disease and on the age distribution of the population under consideration. If the age-specific prevalence is known, the formulas for mean age of onset can be applied directly. Alternatively, the age-specific prevalence inherent in the numbers might be obtained as the solution of a new ODE [6]. As a practical example, the calculations were applied to data on dementia in Germany. The new approach might be beneficial, because studying dementia by empirical studies is very difficult. Characteristics of the age of onset of dementia and the estimated numbers of diseased people in different age groups are highly relevant for health services allocation planning. The methods described here help to predict characteristics of people with chronic diseases (for instance the proportion of people with walking disabilities or in need of care). The methods also allow predictions on regional levels. Because age distributions regionally differ quite substantially, the associated mean ages of onset will be different as well.

## Competing interests

The authors declare that they have no competing interests.

## Authors’ contributions

RB developed the methods, drafted the text, and made the programming. SL proofread the programming. SL and RW critically revised the text, methods, and results. All authors have given important intellectual contributions and final approval of the version to be published.

## Supplementary Material

Additional file 1 Data and source files for use with the statistical software R. Click here for file
